# Intradermally Administered Yellow Fever Vaccine at Reduced Dose Induces a Protective Immune Response: A Randomized Controlled Non-Inferiority Trial

**DOI:** 10.1371/journal.pone.0001993

**Published:** 2008-04-23

**Authors:** Anna H. Roukens, Ann C. Vossen, Peter J. Bredenbeek, Jaap T. van Dissel, Leo G. Visser

**Affiliations:** 1 Department of Infectious Diseases, Leiden University Medical Center, Leiden, The Netherlands; 2 Department of Virology, Erasmus University Rotterdam, Rotterdam, The Netherlands; 3 Department of Research Virology, Leiden University Medical Center, Leiden, The Netherlands; International Vaccine Institute, Republic of Korea

## Abstract

**Background:**

Implementation of yellow fever vaccination is currently hampered by limited supply of vaccine. An alternative route of administration with reduced amounts of vaccine but without loss of vaccine efficacy would boost vaccination programmes.

**Methods and Findings:**

A randomized, controlled, non-inferiority trial was conducted in a Dutch university center between August 2005 and February 2007. A total of 155 primary vaccinated and 20 previously vaccinated volunteers participated. Participants were randomly assigned in a 1∶1 ratio to receive intradermal (i.d.) vaccination with live attenuated yellow fever 17D vaccine at a reduced dose (1/5^th^; 0·1 mL) or the conventional subcutaneous (s.c.) vaccination (0·5 mL). Antibody neutralization titers were determined at 2, 4 and 8 weeks and 1 year after vaccination by counting the reduction in virus-induced plaques in the presence of serial serum dilutions. Adverse events were documented in a 3-week dairy. Viraemia was measured 5 days after vaccination. From 2 weeks up to one year after vaccination, the maximum serum-dilution at which 80% of the virus plaques were neutralized, which indicates protection against yellow fever, did not differ between those given a reduced i.d. dose or standard s.c. dose of vaccine. In all cases the WHO standard of seroprotection (i.e. 80% virus neutralization) was reached (in 77/77 and 78/78, respectively). Similar results were found in the previously vaccinated individuals. Viraemia was detected in half of the primary vaccinated participants, which was not predictive of serological response. In revaccinees no viraemia was detected.

**Conclusions:**

Intradermal administration of one fifth of the amount of yellow fever vaccine administered subcutaneously results in protective seroimmunity in all volunteers. Albeit this vaccination route should enable vaccination of five-times as many individuals at risk for disease, these results should now be confirmed in field studies in areas with potential yellow fever virus transmission to change vaccination policy.

**Trial Registration:**

Nederlands Trial Register ISRCTN46326316

## Introduction

Yellow fever is a re-emerging viral hemorrhagic febrile illness in tropical and sub-tropical areas of Africa and remains a major health threat in South-America. It is estimated to affect 200.000 individuals annually of whom approximately 30.000 die worldwide [1]. The virus is transmitted by infected *Aedes* mosquitoes, and may cause a wide spectrum of disease, from mild symptoms to severe illness accompanied by fever, hepatic and myocardial injury, renal failure, hemorrhage, and even death. There is no curative treatment for yellow fever, making vector control and vaccination essential ingredients in the prevention of yellow fever morbidity and mortality.

Although this flavivirus has never emerged in Asia, the Asian continent is considered vulnerable to future introduction of the virus, because of the presence of a large susceptible human population, the presence of the urban vector and increasing international travel [2]. Also Western countries may be at risk: for instance, in the Netherlands, the *Aedes albopictus* mosquito was introduced via imported bamboo from China, and its capability of transmission of flaviviruses is currently under investigation.

Thus, there is a potential risk for large epidemics of urban yellow fever now that migration of people from rural areas may introduce the virus into areas of high human population density, such as large African and South-American cities. During yellow fever epidemics in non-immune populations, case-fatality rates may be as high as 50% [3]. In case of simultaneous outbreaks in megacities the current emergency stockpile of yellow fever vaccine of 6 million doses will not be sufficient to protect the large populations from the disease [4].

Yellow fever vaccination is the single most important and effective means to prevent the occurrence of yellow fever, and carries a low risk of serious adverse events. The live-attenuated 17D vaccine provides protective immunity within one to two weeks in 95% of those vaccinated [5]. The World Health Organization (WHO) therefore strongly recommends to include yellow fever vaccination in at-risk countries, as part of the routine childhood immunization program. However, hampered by a limited vaccine supply, this recommendation has not yet been acted upon as epidemic emergencies have priority. Besides mass immunization campaigns in response to epidemic outbreaks and planned routine childhood immunization programmes, yellow fever vaccination is used for preventive immunization of travellers to endemic regions [6]. Therefore, to circumvent the consequence of current shortage of vaccine supplies, there is an urgent need to find alternatives for the current standard of yellow fever vaccination, i.e., the subcutaneous administration of 0·5 mL 17D vaccine.

In general, the route of administration of a particular vaccine, e.g., intramuscular, subcutaneous or intradermal, appears to have been reached at arbitrary historical grounds. For the yellow fever vaccine, subcutaneous administration of 0·5 mL followed the initial human trials in which yellow fever 17D (YF−17D) vaccines were first put to extensive use. However, for some vaccines already, for instance rabies, hepatitis B and influenza vaccines, the classical subcutaneous or intramuscular routes have been challenged by the apparent efficacy of intradermal administration using appreciably smaller amounts of vaccine [7]–[10]. The safety and efficacy of this route of administration has not been addressed for the yellow fever vaccine. Interestingly, already in 1943, at the dawn of yellow fever vaccine development, Fox and colleagues observed an immune response after intradermal administration of the YF−17D vaccine [11]. However, the population investigated was small and the method used to assess antibody responses is irreconcilable with current definitions of seroprotection formulated by the WHO. Furthermore, scarification experiments with yellow fever vaccine conducted in the 1950s revealed a lower response rate when compared to subcutaneous inoculation [12], [13].

In this study we investigate the efficacy of intradermal inoculation of yellow fever vaccine at one fifth the amount given subcutaneously, as a potential strategy to reduce costs and increase vaccine coverage in areas with limited amounts of vaccine available for mass vaccination as well as for travellers to these areas. Furthermore, to elucidate requirements for the induction of an effective immune response to yellow fever vaccination we assessed antibody responses in relation to post-vaccination viraemia in both primary and revaccinated individuals.

## Methods

The protocol for this trial and supporting CONSORT checklist are available as supporting information; see checklist S1 and protocol S1 with amendments S2, S3 and S4.

### Objectives

This study was conducted to determine whether reduced dose i.d. yellow fever vaccination (1/5^th^; 0·1 mL) would be as efficacious and safe as the conventional s.c. vaccination (0·5 mL). Efficacy of vaccination was measured by virus neutralization plaque reduction assay.

### Study design and Participants

Healthy volunteers of 18 years and older were eligible for inclusion. We excluded volunteers with a compromised immunity due to underlying illness or immunosuppressive medication, pregnant volunteers and those with chicken egg allergy. The study was carried out between August 2005 and February 2007. Subjects were randomly assigned by the investigator (AHR) to either receive intradermal (i.d.) (experimental vaccination group) or subcutaneous (s.c.) (conventional vaccination group) yellow fever vaccination. Randomization was performed with the use of sealed envelopes containing the vaccination code balanced through in permuted blocks of 10 each. Vaccinations were administered at the travel clinic of the LUMC by the investigators who were trained in both methods of vaccine administration.

In the experimental vaccination group, participants received 0·1 mL YF−17D vaccine intradermally on the dorsal side of the right forearm. The syringe which was used for i.d. administration is identical to the syringe used for administration of tuberculin in the Mantoux test. The quality of the i.d. injection was defined by the diameter of the arisen cutaneous wheal (adapted from the tuberculin skin test) [14], with 6 mm being the lowest acceptable diameter. The conventional vaccination group received 0·5 ml YF−17D vaccine subcutaneously in the right upper deltoid region.

#### 17D Yellow Fever Vaccine

The live, attenuated, 17D vaccine used in this study was manufactured on embryonated chicken eggs according to WHO regulations and stored according to manufacturer's guidelines. All administered vaccines originated from the same vaccine lot (Stamaril, Lot no Y5597, Sanofi Pasteur, France). A single vaccination dose of 0·5 ml contained approximately 3·5×10^4^ plaque forming units (PFU), measured in two randomly selected vials. Multiple dosages (maximally 4) were obtained from one vial for i.d. vaccination. After reconstitution, vials were stored at 4°C and discarded after maximally 4 hours.

### Procedures

#### Data collection

At the time of inclusion, data on demographic and clinical characteristics of the participants were obtained, including information on possible flavivirus exposure (defined as travel to a flavivirus endemic country) in the 5 years prior to entering the study and previous yellow fever vaccination. Blood samples were collected in all (155) primary vaccinated participants before vaccination, and 4 and 8 weeks after vaccination. An additional blood sample was collected 2 weeks after vaccination in 55 primovaccinees (the last 55 consecutive subjects entering the study) to investigate the kinetics of the neutralizing antibody response in more detail.

Extra ethylenediamine tetraacetic acid (EDTA) blood samples were collected 5 days after vaccination in the first 24 consecutive primovaccinees entering the study.

**Figure 1:**
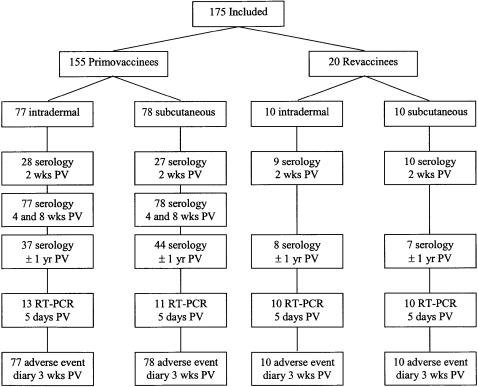
Flow chart of study participants. Included study participants from August 2005 until February 2007. PV = post vaccination. RT−PCR = Reverse transcriptase polymerase chain reaction. Wks = weeks and yr = year.

In 20 previously vaccinated participants blood was drawn before vaccination, and 5 days and 2 weeks after booster vaccination (figure 1). Approximately one year after vaccination, one additional blood sample was taken from all participants who could be contacted (96 participants). A financial compensation was given for every blood sample collection at completion of the study. None of the participants withdrew prematurely.

Participants were asked to document clinical symptoms (local and systemic) after vaccination in a three-week diary. Solicited symptoms were; erythema, pain and swelling at the site of injection, fever and myalgia. Severity of adverse events was documented as – (absent), +/− (mild), + (moderate) and ++ (severe).

#### Constant virus – varying serum dilution Plaque Reduction Neutralization Test (PRNT)

The tests were carried out in 6-well plates (Corning Inc., USA) using a slightly modified technique described originally by De Madrid and Porterfield [15]. Briefly, approximately 6×10^5^ Vero cells/mL were seeded per well in 6−well plates and cultured to obtain a confluent monolayer. Sera were complement inactivated at 56°C for 1 hour. Prevaccination sera were tested in 1∶16 dilution, to which 100 PFU of 17D-YF were added. Postvaccination sera were tested in two-fold dilutions starting from 1∶16 to 1∶512. 100 PFU YF−17D virus were added to each serumdilution. All test sera were assayed in duplicate. After 1 hour incubation on ice, the mixtures of virus and serum were added to the Vero cell monolayers and incubated for 1 hour at 37°C. An overlay of 2× DMEM and 2% agarose was added. After 5 days of incubation at 37°C, the overlay was discarded and cell monolayers were stained with crystal violet. Plaques were counted by eye. Virus neutralization (VN) was calculated for each serum dilution (i) with the following formula: VN(i) = 100× (number of PFU in diluted postvaccination serum/number of PFU in pre-vaccination serum (in a 1∶16 dilution)). For comparison of i.d. and s.c. vaccination, serum dilution at which log_10_ neutralization index 0·7 (80% VN) occurred was taken as endpoint, as this corresponds to the generally accepted definition of protection [16].

#### Reverse Transcriptase-Polymerase Chain Reaction (RT-PCR)

RT−PCR of YF−17D was performed at the department of Virology of the Erasmus Medical Center according to Nijhuis and colleagues [17]. Briefly, viral RNA was isolated and reverse transcribed (Taqman Reverse Transcription Reagents, Applied Biosystems International). cDNA synthesis was performed in a J Mini Gradient Thermal Cycler (BioRad, Netherlands) for real-time PCR, the following YF specific primers and probe were used [18]:

YFV-1 (forward) AATCGAGTTGCTAGGCAATAAACAC
YFV-2 (reverse) TCCCTGAGCTTTACCAGA
YFV-P (probe) FAM-ATCGTTGAGCGATTAGCAG-BHQ

with FAM (6−carboxyfluorescein) as 5′-reporter dye and BHQ (Black Hole Quencher) as the 3′-quencher dye. Real-time PCR was monitored on ABI Prism 7500 Seq. Detection System (Applied Biosystems International). Cycle threshold (Ct) values were used to compare viraemia in i.d. and s.c. groups quantitatively.

### Ethics

The protocol and consent forms were approved by the Medical Ethical Committee of the Leiden University Medical Center (LUMC), the Netherlands (ISRCTN46326316). Written informed consent was obtained from each participant.

### Statistical methods

Power calculations for primovaccinees were based on a one-sided non-inferiority according to Armitage P., et al. [19], formula 18.5, with a maximally acceptable difference (δ) of 0·04 between the experimental and conventional vaccination group, α of 0·05, β of 0·2 and a π (overall probability of positive response) of 0·99 [5], which makes σ^2^ = 0·0099. The number of participants needed to confirm non-inferiority of low dose i.d. vaccination under these assumed conditions are 77 per group. For the antibody response in previously vaccinated individuals receiving a booster vaccination, basic descriptive statistics are used. It was anticipated that the small number in this subgroup would not allow a definite conclusion concerning non-inferiority and no power calculation was performed. Twenty previously vaccinated persons were included to monitor possible trends in interference of neutralizing antibodies in yellow fever vaccination. Paired t-test was performed to calculate their increase in neutralization after booster vaccination and linear regression was used to calculate influence of circulating antibodies on booster vaccination. Neutralizing capacity of sera after i.d. and s.c. vaccination were compared with Student's t-test. Where appropriate, Chi-square tests were used, and Wilcoxon's test for non-parametrical distributed numerical data. Statistical analysis was performed using a computer-assisted software package (SPSS version 12.0, SPSS Inc., Chicago, IL).

## Results

### Study population

We enrolled 175 volunteers from August 2005 to February 2007 (figure 1). Baseline characteristics of the study population are given in table 1.

**Table 1:** Comparability of intradermally and subcutaneously vaccinated groups

Participants		Vaccine administration		p-value
		Intradermal	Subcutaneous	
Primovaccinees (N = 155)	N Female (%)	56 (73)	65 (83)	0·1
	Mean age (range)	27 (18–61)	25 (19–70)	0·2
	Flavivirus$ N yes (%)	33 (43)	26 (33)	0·3
Revaccinees (N = 20)	N Female (%)	7 (70)	8 (80)	-
	Mean age (range)	30 (20–50)	34 (21–48)	0·4

Age and gender distribution in primary (77 i.d., 78 s.c.) and booster (10 i.d., 10 s.c.) vaccinated populations. YF-17D = yellow fever vaccine virus.

$ Flavivirus = possible flavivirus encounter in past five years defined as travelled to flavivirus endemic destination.

Concerning the accuracy of i.d. vaccine delivery, the mean diameter of the cutaneous wheal measured after vaccination was 8 mm (range 6–10 mm), indicating that all (N = 87) i.d. vaccination wheals met the minimal requirement for acceptable size.

### Vaccine efficacy

**Figure 2:**
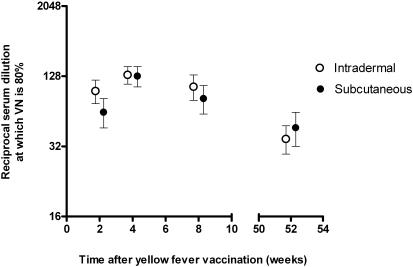
Protective virus neutralization after intradermal or subcutaneous vaccination against yellow fever. Comparison of reciprocal serum dilutions at which 80% of yellow fever virus is neutralized in constant virus – varying serum dilution test after intradermal and subcutaneous YF vaccination in primary vaccinated participants (n = 155). Bars represent 95% Confidence Intervals (CI). Virus neutralizing capacity of serum in both groups was performed at similar time points but indicators are juxtaposed for visual enhancement. VN = virus neutralization.

Four weeks after vaccination, 80% virus neutralization (VN) at the least diluted serum (dilution of 1∶16) was achieved by 77 of 77 of the intradermally and by 78 of 78 of the subcutaneously vaccinated primovaccinees. The percentage of VN in both study groups was linearly correlated to serum dilution at all time points measured (data not shown). Ninety percent neutralization was achieved by 70 of 77 (91%) and by 69 of 78 (89%), respectively. Plotting of neutralization indices against serum dilution showed similar kinetics of i.d. and s.c. vaccination at all measured time points (data not shown). This allowed us to compare the serum dilution at which 80% of yellow fever was neutralized, which is similar in both groups at all time points measured (figure 2).

No difference in percentage of virus neutralization was measured in either (i.d. or s.c.) group between male and female participants, nor between recent travel to flavivirus endemic countries or not (data not shown).

Neutralizing capacity of 1∶16 diluted prevaccination serum of previously vaccinated participants ranged from 2% to 97% reflecting the wide range of years since their last YF vaccination (0·5 to 18 years). The mean percentage of VN by the least diluted serum before vaccination in the i.d. group was 77% (range 51%–97%) and in the s.c. group was 74% (range 2%–97%). All revaccinees reached protective neutralization immunity 2 weeks (19/19) and 1 year (15/15) after vaccination.

**Figure 3:**
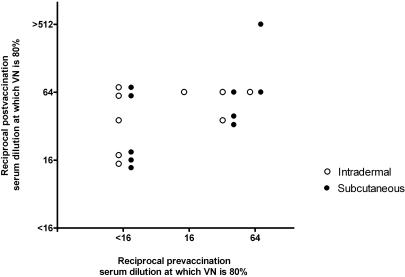
Pre- and post vaccination virus neutralizing capacity of serum of previously vaccinated participants. Pre- and postvaccination (2 weeks) serum dilutions at which 80% VN occurred in previously vaccinated participants. When 80% VN was not reached by the least diluted serum (1∶16), samples were defined as <16 (reciprocal serum dilution). VN = Virus neutralization.

Both the i.d. and the s.c. group of revaccinated participants showed a significant rise in VN after booster vaccination. The mean increase in percentage of neutralization by serum (dilution 1∶16) before and 2 weeks after vaccination in the i.d. vaccinated participants was 18% (95% CI; 8%–28%) and 20% (95% CI; 4%–36%) in the s.c. group (data not shown). To investigate the influence of prevaccination neutralizing antibody titer on postvaccination VN, pre- and post vaccination serum dilutions at which 80% VN occurred were plotted (figure 3). In linear regression analysis, an increase in postvaccination VN correlated significantly with a higher prevaccination antibody titer (coefficient 0.54, p = 0·02). Thus, the presence of circulating neutralizing antibodies in this population did not inhibit a booster response.

Viraemia was measured by RT-PCR 5 days after vaccination in 24 primovaccinees and all revaccinees (N = 20). In the latter no YF-17D RNA was detected in the blood. The percentage of primary vaccinated subjects positive for YF virus detection was comparable in the i.d. (7 of 13, 54%) and s.c. (5 of 11, 45%) group, as were the mean Cycle threshold (Ct) values (35·86 cycles and 37·52 cycles, respectively).

**Figure 4:**
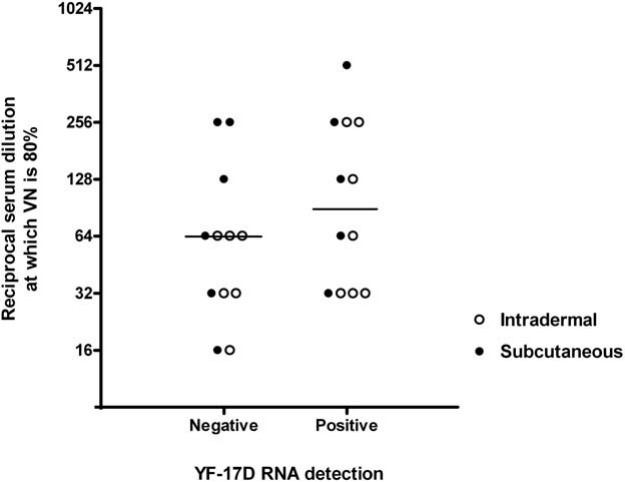
Virus neutralizing capacity of YF-RNA negative and positive sera. Comparison of reciprocal serum dilutions, of serum obtained 4 weeks after vaccination, at which 80% VN occurred between positive and negative YF-17D RNA detection by RT-PCR in primary vaccinated participants (N = 24). Bars represent the median reciprocal serum dilution. VN = Virus neutralization.

No difference was measured in the serum dilution at which 80% VN occurred 4 weeks after vaccination between those with and those without viraemia, irrespective of the route of vaccine administration (figure 4).

### Vaccine safety

Participants reported duration and severity of adverse events after yellow fever vaccination in a 3−week diary. In primary vaccinated participants i.d. vaccination evoked redness and swelling at the site of inoculation more frequently and for a significantly longer period than after s.c. vaccination (p<0·001). Itching at the site of injection was also reported more by i.d. vaccinated primovaccinees (p = 0·02). The s.c. vaccinated primovaccinees reported significantly longer pain at the site of injection (p = 0·03), and more s.c. primary vaccinated participants reported myalgia (p<0·01) (table 2). In previously vaccinated participants, a similar trend of adverse events was monitored except for myalgia.

**Table 2:** Solicited adverse events after primary and booster YF-17D vaccination.

Adverse event			Primary vaccination (N = 155)		Booster vaccination (N = 20)	
			Intradermal	Subcutaneous	Intradermal	Subcutaneous
Local	Erythema	N yes (%)	63 (82)	25 (32)	6 (60)	1 (10)
		Mean N days (s.e.m.)	4·3 (±0·5)	1·1 (±0·2)	3·2 (±1·0)	1·0 (±0·9)
	Swelling	N yes (%)	52 (68)	9 (12)	6 (60)	0 (0)
		Mean N days (s.e.m.)	2·6 (±0·4)	0·3 (±0·1)	2·6 (±0·9)	-
	Pain	N yes (%)	6 (8)	15 (19)	2 (20)	0 (0)
		Mean N days (s.e.m.)	0·1 (±0·06)	0·6 (±0·2)	0·2 (±0·1)	-
	Severity	N +/− (mild)	39	15	3	-
		N + (moderate)	24	9	2	2
		N ++ (severe)	-	1		-
Systemic	Myalgia	N yes (%)	12 (16)	27 (22)	1 (10)	1 (10)
		Mean N days (s.e.m.)	0·4 (±0·1)	0·7 (±0·1)	0·1 (±0·1)	0·7 (±0·7)
	Fever	N yes (%)	4 (5)	8 (10)	0 (0)	0 (0)
		Mean N days (s.e.m.)	0·1 (±0·03)	0·2 (±0·06)	-	-
	Severity	N +/− (mild)	9	17	-	-
		N + (moderate)	3	8	1	1
		N ++ (severe)	-	2	-	-

Safety of vaccination expressed in various parameters. Severity of adverse events could be graded with - (absent), +/− (mild), + (moderate) and ++ (severe). S.e.m. = standard error of the mean.

The severity of adverse events due to vaccination, which was reported on a 4-level scale (−, +/−, +, ++), did not reveal a difference in experienced discomfort (both local and systemic) between the i.d. and s.c. group. Of the reported adverse events, 2/3^rd^ was experienced as mild (+/−) and 1/3^rd^ as moderate (+). No i.d. vaccinated and 3 s.c. participants rated their events as severe (++).

## Discussion

Intradermal administration of 1/5^th^ of the conventional yellow fever vaccine dose was non-inferior to standard subcutaneous vaccination of the full dose as far as protective immune response and safety is concerned: at 2, 4 and 8 weeks after administration, as well as one year later, the titers of yellow fever-neutralizing antibodies were identical in individuals being primary vaccinated intradermally or subcutaneously. Both i.d. and s.c. administration of the vaccine resulted in protective seroimmunity in all subjects. Finally, the kinetics of the immune response were similar in both groups with neutralizing antibody responses peaking at 4 weeks after vaccination.

Several aspects of this study require comment. First, assuming 99% seroprotection after primary vaccination in both groups, the population size in this study does not allow to detect differences less than 4% between the experimental (i.d.) and conventional (s.c.) vaccination groups. However, the numbers are sufficient to reliably measure a log 0·7 virus neutralizing capacity in at least 95% of those vaccinated intradermally, which meets the minimal required percentage of seroprotection after vaccination, as defined by the WHO [20]. Second, the viral dose contained in the trial vaccine was 3·5×10^4^ PFU/0·5mL, which is equivalent to approximately 5×10^3^ Mouse Lethal Dose (MLD)_50_ (21). A fivefold reduction of vaccine dose for i.d. delivery then still contains the minimal potency requirement (1×10^3^ MLD_50_) as defined by the WHO [20], meaning that the results of this study cannot exclude that s.c. vaccination with 0·1 mL dose might be protective. Several lines of evidence however suggest that this may not be the case. More than sixty years ago Fox and colleagues verified the protective efficacy of human serum from vaccinees in a mouse challenge model and observed that at a similar vaccine dose, sera from intradermally injected subjects were more efficacious than sera of those injected subcutaneously [11]. Additionally, 0·1 mL s.c. delivery of a live attenuated chimeric flavivirus vaccine against Japanese encephalitis in non-human primates resulted in a 7−fold lower neutralizing antibody response compared to 0·1 mL i.d. delivery by micro needle [22]. Finally, this study has been performed in healthy adult volunteers who represent travellers to and not individuals living in an area of potential yellow fever transmission. This study should be repeated in a population living in a yellow fever endemic area, to account for differences in skin tissue composition, possible interactions by cross-reactive antibodies against other flaviviruses, and possible decreased immune response due to malnutrition or chronic parasitic infections.

In regard to the reproducibility of these results, the significant variation in viral load between YF-17D vaccine batches is of importance. The batches generally contain 5–50 times the minimal required potency dose to account for possible loss during storage and transportation [5]. The YF-17D batch used in this study contained five times the minimal required potency dose, and is therefore at the low side of the batch-variability in viral vaccine load. Intradermal YF-17D vaccination with other batches will thus yield similar results, as no other batch is likely to contain less virus particles.

Correct i.d. vaccination is technically more demanding than subcutaneous or intramuscular vaccination. By introducing a minimal diameter cutoff of the cutaneous wheal following i.d. vaccination, we allowed to control for proper i.d. delivery of the vaccine. To our opinion, this simple test is a valuable tool to ensure correct i.d. vaccination.

Local adverse events such as erythema and swelling were reported to occur longer in the i.d. vaccinated group. This is consistent with other intradermally administered vaccines [7], [8], and might represent the inflammatory reaction due to activation of local immunomodulating cells. To our opinion this increased duration of local adverse events will not be a reason to renounce the new cost-effective method of yellow fever vaccination investigated, as they were not experienced as more severely than the adverse events in the s.c. group. Evidently, adverse events with a frequency beneath 1/77 after low dose i.d. vaccination could not be detected in this study.

The participants who had been previously vaccinated against yellow fever all showed seroprotection after booster vaccination, irrespective of their pre-booster VN capacity, implying that circulating neutralizing antibodies did not interfere with the induction of a booster response. Furthermore, this study shows that detectable YF−17D replication as evidenced by viraemia in the week after vaccination, was not required for induction of a booster response, which is consistent with previous findings by Reinhardt et al. [23].

The enhanced efficiency of the i.d. route of vaccination may be explained by direct targeting of antigen presenting cells (APCs) in the dermis and epidermis. Despite the possibility of YF−17D replication in dendritic cells [24], Palmer and colleagues found this replication to be restricted due to rapid processing of the virus [25]. Together with the fact that despite the lower vaccine dose the number of intradermally vaccinated participants in which viraemia was measured was not reduced, it is likely that an even more reduced vaccine dose (than fivefold reduction) administered i.d. could induce a protective immune response.

The findings of this study have the following practical implications: 1) in case of an outbreak of urban yellow fever or vaccine shortage for travellers to endemic areas, i.d. administration of yellow fever vaccine will allow immunization of at least four times as many individuals as s.c. vaccination with the same limited vaccine supply, 2) provided that these results can be confirmed in field studies in areas with potential yellow fever virus transmission, the i.d. vaccination strategy could be implemented in routine immunization programmes and support the ‘yellow fever risk reduction initiative’ launched by the WHO and UNICEF to envisage the immunization of 48 million people in 12 high-risk countries between now and 2010 [26], 3) finally, these results suggest that travellers with a possible history of egg allergy in whom an i.d. test dose of 0·1 ml YF−17D vaccine yielded a strong local urticarial reaction do not need further vaccination, but this should always be checked by virus neutralization tests.

## Supporting Information

Checklist S1 CONSORT Checklist
(0.07 MB DOC)

Protocol S1 Trial Protocol
(0.10 MB DOC)

Protocol S2 Trial Protocol
(0.03 MB DOC)

Protocol S3 Trial Protocol
(0.03 MB DOC)

Protocol S4 Trial Protocol
(0.03 MB DOC)
