# Supplementary material for: Intradermally Administered Yellow Fever Vaccine at Reduced Dose Induces a Protective Immune Response: A Randomized Controlled Non-Inferiority Trial
Source: PLoS One. 2008 Apr 23;3(4):e1993. doi: 10.1371/journal.pone.0001993 (PMC2297511; doi:10.1371/journal.pone.0001993)
Supplement: Protocol S1 — Trial Protocol (0.10 MB DOC) [file pone.0001993.s002.doc]

PROTOCOL P05.059

Titel: Gele koorts vaccinatie: vergelijking tussen effectiviteit van subcutane en intracutane injectie

drs. A.H.E. Roukens, AIO, dr. L.G. Visser, hoofdonderzoeker, prof.dr. J.T. van Dissel

Afdeling Infectieziekten (C5-P)

Leids Universitair Medisch Centrum

+31 71 5262613

L.G.Visser@lumc.nl

Protocol P05.059

Versie 2.0

9 juni 2005

# Inhoudsopgave

[Inhoudsopgave 2](#__RefHeading___Toc106092102)

[Samenvatting protocol 3](#__RefHeading___Toc106092103)

[1. Inleiding 4](#__RefHeading___Toc106092104)

[2. Vraagstelling 5](#__RefHeading___Toc106092105)

[3. Doel 5](#__RefHeading___Toc106092106)

[4. Proefpersonen 5](#__RefHeading___Toc106092107)

[5. Onderzoeksopzet 5](#__RefHeading___Toc106092108)

[6. Privacy 7](#__RefHeading___Toc106092109)

[7. Bijwerkingen 7](#__RefHeading___Toc106092110)

[8. Statistische analyse 7](#__RefHeading___Toc106092111)

[9. Implicaties 8](#__RefHeading___Toc106092112)

[10. Onafhankelijke arts 8](#__RefHeading___Toc106092113)

[11. Verzekering 8](#__RefHeading___Toc106092114)

[12. Financiering 8](#__RefHeading___Toc106092115)

[13. Verslaglegging 8](#__RefHeading___Toc106092116)

[Referenties 16](#__RefHeading___Toc106092121)

# Samenvatting protocol

Gele koorts vaccinatie: vergelijking tussen effectiviteit van subcutane en intracutane injectie

Regelmatig is er een dreigend tekort aan gele koorts vaccin, waardoor de vraag rijst of er andere, even effectieve, methoden zijn om te vaccineren tegen gele koorts waarbij minder vaccin nodig is. Uit twee recent gepubliceerde onderzoeken blijkt dat vaccinatie tegen het influenzavirus in een kleine dosis en door middel van intracutane injectie even effectief is als intramusculaire injectie met de normale dosis (Belshe et al. 2286-94;Kenney et al. 2295-301). In dit protocol gaan we na of hetzelfde geldt voor het gele koorts vaccin.

De proefpersonen waarbij we de effectiviteit gaan meten zijn gezonde vrijwillgers, representatief voor de groep reizigers die in aanmerking komen voor het vaccin vanwege hun reisbestemming. Eén groep zal het gele koorts vaccin intracutaan en in een vijfde van de normale dosis toegediend krijgen, de andere groep de normale dosis subcutaan. Vergelijking tussen de antistoftiters van beide groepen zal uitwijzen of de intracutane injectie een even goede bescherming biedt.

Indien blijkt dat een kleinere dosis van het gele koorts vaccin indien intracutaan toegediend even effectief is om bescherming te bieden, zou dit onderzoek een voorlopige oplossing voor het dreigende tekort aan het vaccin bieden.

**Titel: Gele koorts vaccinatie: vergelijking tussen effectiviteit van subcutane en intracutane injectie**

# 1. Inleiding

Gele koorts vaccin is een levend verzwakt vaccin. Afgelopen twee jaar is er door fouten in het productieproces, drie maal een tekort aan gele koorts vaccin ontstaan. Onlangs zijn twee onderzoeken gepubliceerd waaruit blijkt dat vaccinatie tegen het influenzavirus even effectief is bij intracutane, in plaats van intramusculaire injectie (Belshe et al. 2286-94;Kenney et al. 2295-301). Volgens de onderzoeken naar intracutane influenzavaccinatie is een 2,5 tot 5 maal kleinere dosis van het vaccin voldoende om dezelfde respons op te wekken als de tot nu toe uitgevoerde vaccinatie door middel van intramusculaire injectie. De mogelijke verklaring hiervoor is, dat de huid meer antigeenpresenterende cellen bevat dan de spier en subcutaan weefsel, en dus een betere immuunrespons kan opwekken (Steinman and Pope 1519-26). Dit principe zou mogelijk ook van toepassing kunnen zijn op levend verzwakte vaccins, zoals het gele koorts vaccin.

De WHO heeft de minimaal vereiste hoeveelheid virus in gele koorts 17D vaccin op 3.0 log MLD_50_, gesteld (WHO, Requirements for Yellow Fever Vaccination). Bij deze dosis vertoont 90% van de geïmmuniseerden een beschermende antistoftiter. In het gele koorts 17D vaccin dat gebruikt wordt voor vaccinatie zit een vijf- tot vijftigvoud van deze vereiste hoeveelheid. De reductie van de experimentele dosis van 0.5 naar 0.1 ml impliceert dus dat hiermee minstens de minimaal vereiste dosis voor bescherming wordt gebruikt.

Eén keer eerder in de geschiedenis van de ontwikkeling van het gele koorts vaccin is nagegaan of er verschil in effectiviteit bestond tussen de verschillende toepassingswegen (Fox et al., 1943). Hierbij werd geconstateerd dat de intracutane toedieningsweg bij eenzelfde dosis effectiever was dan de subcutane weg. Dit verschil in immuunsrespons verdwijnt wanneer een voldoende grote dosis subcutaan wordt toegediend.

Indien uit dit onderzoek blijkt dat bescherming na intracutane injectie met een kleinere dosis van het gele koorts vaccin even effectief is, zou de intracutane toedieningsweg een oplossing bij het dreigende tekort aan het vaccin kunnen bieden.

# 2. Vraagstelling

Biedt intracutane injectie van het gelekoortsvaccin met een vijfde van de dosis dezelfde bescherming als vaccinatie via de subcutane weg met volledige dosis.

Heeft de intracutane injectie meer, minder of andere bijwerkingen heeft dan de subcutane injectie van het vaccin.

Gaat intracutane vaccinatie gepaard met dezelfde (mate van) viremie als na subcutane vaccinatie.

# 3. Doel

Het doel van dit onderzoek is om bij een dreigend tekort aan gele koorts vaccin via de intracutane toedieningsweg meer personen te kunnen immuniseren met dezelfde hoeveelheid subcutaan vaccin.

# 4. Proefpersonen

De effectiviteit van de intracutane injectie van het gele koorts vaccin willen we bepalen bij gezonde volwassen vrijwilligers ouder dan 18 jaar, die representatief zijn voor de groep reizigers die in aanmerking komt voor het vaccin vanwege hun reisbestemming. De proefpersonen worden geworven door middel van advertenties in het LUMC.

Zowel proefpersonen met als zonder een gele koorts vaccinatie in hun voorgeschiedenis worden geïncludeerd.

Om de volgende redenen worden personen uitgesloten van deelname; chloroquine gebruik, zwangerschap, een medische conditie die de afweer ernstig vermindert (beschreven in de landelijke protocollen voor reizigersadvisering), zoals; maligniteiten, diabetes mellitus en gebruik van immuunsuppressieve medicatie waaronder corticosteroiden en cytostatica. Bij onduidelijkheid over exclusie wordt overlegd met dr. L.G. Visser.

Deelname aan het onderzoek zal plaatsvinden na telefonische informatieverstrekking aan de proefpersoon en het verkrijgen van een toestemmingsverklaring bij het maken van een afspraak. Bij bezoek aan de vaccinatiepolikliniek wordt de toestemmingsverklaring ondertekend.

# 5. Onderzoeksopzet

Randomised controlled trial: proefpersonen worden door middel van een willekeurige elektronische nummertoewijzing gerandomiseerd in een groep die het gele koorts vaccin intracutaan toegediend krijgt, de cases, en een even grote groep die het vaccin subcutaan toegediend krijgt, de controles.

Vóór de toediening van het vaccin wordt bloed afgenomen, hieruit wordt de antistoftiter tegen het gele koorts virus bepaald (nulserum).

Bij het verlaten van de polikliniek krijgt de proefpersoon een dagboekje mee waarin bepaalde aspecten van de plaats van injectie en van de algehele gezondheidstoestand genoteerd kunnen worden. Vier en acht weken na de toediening van de primaire vaccinatie en twee weken na revaccinatie (Reinhardt et al. 159-67) vindt nogmaals bloedafname plaats. Hieruit wordt de antistoftiter bepaald tegen het gele koorts vaccin die vervolgens wordt vergeleken met de titer vóór vaccinatie. Hierna worden de antistoftiters van de cases vergeleken met die van de controles, evenals de bijwerkingen die op de polikliniek zijn geobjectiveerd of die in het dagboekje genoteerd zijn. Antistoftiters worden bepaald door middel van neutralisatietests en ELISA (enzyme linked immunosorbent assay).

Bij een subgroep van 20 proefpersonen voor primovaccinatie en alle proefpersonen voor revaccinatie wordt op dag 5 bloed afgenomen ter bepaling van de viremie (zie tabel 1 en 2).

| **Primovaccinatie** | **tijd (dagen)** | | | |
| --- | --- | --- | --- | --- |
|  | **0** | **5** | **28** | **56** |
| gele koorts vaccinatie (intra- of subcutaan) | x |  |  |  |
| bloedafname voor 380 proefpersonen | x |  | x | x |
| bloedafname voor 20 proefpersonen | x | x | x | x |

*tabel 1. Schema bloedafname gele koorts primovaccinatie*

| **Revaccinatie** | **tijd (dagen)** | | |
| --- | --- | --- | --- |
|  | **0** | **5** | **14** |
| gele koorts vaccinatie (intra- of subcutaan) | x |  |  |
| bloedafname voor 40 proefpersonen | x | x | x |

*tabel 2. Schema bloedafname gele koorts revaccinatie*

De kwaliteit van intracutane injectie wordt beoordeeld op de diameter van de kwaddel die hierbij ontstaat, een objectieve maat voor de hoeveelheid vaccin die in de huid geïnjecteerd wordt.

Alle handelingen met uitzondering van de antistoftiterbepaling vinden plaats op de vaccinatiepolikliniek van het Leids Universitair Medisch Centrum. De antistoftiterbepaling en viremie meting wordt uitgevoerd door het Erasmus ziekenhuis te Rotterdam.

Indien geen antistoffen kunnen worden aangetoond, wordt na afname van een nieuw serummonster subcutaan gevaccineerd en zonodig opnieuw antistoftiter na vier weken bepaald.

# 6. Privacy

De medische gegevens van de proefpersonen die voor dit onderzoek door de verantwoordelijke onderzoekers verzameld worden, vallen onder het medisch beroepsgeheim. De uitkomsten van het onderzoek zullen alleen anoniem worden gepubliceerd en zijn nooit te herleiden tot de individuele proefpersonen. Behalve de onderzoeksverantwoordelijken en medewerkers aan wie door deze verantwoordelijken toestemming is verleend, heeft niemand inzage in de gegevens van de proefpersonen. De te onderzoeken samples worden gecodeerd naar het Erasmus MC te Rotterdam gestuurd, waarbij de coderingssleutel in het LUMC blijft, onder toezicht van de verantwoordelijke onderzoekers.

# 7. Bijwerkingen

Bijwerkingen die kunnen optreden bij gele koorts vaccinatie zijn:

1. lokale reacties zoals roodheid en zwelling op de injectieplaats
2. systemische reacties zoals spierpijn en lichte koorts

Zelden treden de volgende bijwerkingen op: allergische reacties, hoofdpijn, jeuk, huiduitslag, urticaria, geelzucht, bursitis, neuritis en lymfadenopathie.

# 8. Statistische analyse

Om aan te tonen dat er geen statistisch verschil is tussen de antistofrespons na intracutane en na subcutane primaire vaccinatie gaan we uit van een totale populatie van 400 proefpersonen, 200 cases en 200 controles (zie bijlage 1). Met deze omvang van de onderzoekspopulatie kan bij een α van 0.05 en een power van 80% de equivalentie (in dit onderzoek non-inferioriteit gezien het eenzijdige betrouwbaarheidsinterval) aangetoond worden tussen de verschillende toedieningswegen. Stel hierbij dat subcutane toediening 99% en intracutane toediening een 99% seroprotectie genereren, met een eenzijdige ondergrens van het betrouwbaarheidsinterval, 90%, wat de minimaal vereiste bescherming is volgens de WHO. Omdat deze verwachte uitkomsten onbekend zijn zal een tussentijdse evaluatie uitgevoerd worden als data verzameld zijn van 50 controles en 50 cases, en vervolgens van 100 controles en 100 cases.

Omdat er een schaarste aan gepubliceerde gegevens is over de viremie en de antistofrespons na revaccinatie wordt bij een groep van twintig proefpersonen per toedieningsweg gevaccineerd waarbij deze viremie en respons worden gemeten.

Om een populatiegrootte te berekenen waarmee een verschil aangetoond kan worden tussen viremie na de verschillende toedieningswegen wordt eerst een pilotstudie uitgevoerd. Aan deze pilotstudie zullen zowel in de primovaccinatie- als in de revaccinatiegroep twintig proefpersonen deelnemen.

# 9. Implicaties

Bevindingen uit dit onderzoek kunnen implicaties hebben voor alle vaccinatieklinieken. Waar het nu nog de gewoonte is om het gele koorts vaccin subcutaan te injecteren zou bij positieve resultaten uit dit onderzoek de intracutane injectie van het vaccin op alle vaccinatieklinieken ingevoerd kunnen worden. Het voordeel daarbij zou zijn dat een lagere dosis vaccin vereist is om dezelfde bescherming te bieden als de huidige methode, en er dus meer personen tegen gele koorts beschermd kunnen worden.

Ook kan dit onderzoek de bevindingen van de onderzoeken van Belshe en Kenney ondersteunen en toetsen of deze observatie ook voor levend verzwakte vaccins geldt.

# 10. Onafhankelijke arts

De onafhankelijke arts in dit onderzoek is dr. F.P. Kroon, internist-infectioloog in het Leids Universitair Medisch Centrum.

# 11. Verzekering

Gezien de belasting voor de proefpersoon (eenmaal intracutane of subcutane vaccinatie en drie- tot viermaal bloedafname door gekwalificeerd persoon) wordt een verzekering afgesloten.

# 12. Financiering

Dit onderzoek wordt gefinancierd door de afdeling Infectieziekten van het Leids Universitair Medisch Centrum.

# 13. Verslaglegging

Publicatie van de resultaten uit dit onderzoek zal volgens de volgende auteursvolgorde geschieden: A.H.E.Roukens, A.C.T.M.Vossen, J.T.van Dissel, L.G.Visser

# Referenties

Belshe, R. B. et al. "Serum antibody responses after intradermal vaccination against influenza." N.Engl.J.Med. 351.22 (2004): 2286-94.

Kenney, R. T. et al. "Dose sparing with intradermal injection of influenza vaccine." N.Engl.J.Med. 351.22 (2004): 2295-301.

Reinhardt, B. et al. "Development of viremia and humoral and cellular parameters of immune activation after vaccination with yellow fever virus strain 17D: a model of human flavivirus infection." J.Med.Virol. 56.2 (1998): 159-67.

Steinman, R. M. and M. Pope. "Exploiting dendritic cells to improve vaccine efficacy." J.Clin.Invest 109.12 (2002): 1519-26.

Fox, J.P. et al. “Field studies on the immune response to 17D yellow fever virus.”

Am. J. Hygiene 38.2 (1943): 113-138.

World Health Organization, Requirements for Yellow Fever Vaccine. Addendum

1987. World Health Organization Technical Report Series N° 771. Annex 9.

Geneva, WHO, 1988, p 208.
