# Supplementary material for: Intradermally Administered Yellow Fever Vaccine at Reduced Dose Induces a Protective Immune Response: A Randomized Controlled Non-Inferiority Trial
Source: PLoS One. 2008 Apr 23;3(4):e1993. doi: 10.1371/journal.pone.0001993 (PMC2297511; doi:10.1371/journal.pone.0001993)
Supplement: Protocol S2 — Trial Protocol (0.03 MB DOC) [file pone.0001993.s003.doc]

**Amendement**

**Protocol P05.059**

20 november 2006

Titel: Gele koorts vaccinatie: vergelijking tussen effectiviteit van subcutane en intracutane injectie

Dit amendement betreft een verzoek om het goedgekeurde onderzoeksprotocol (P05.059, versie 2, 9 juni 2005) uit te breiden.

In het amendement van 19 oktober 2006 staat beschreven dat we voor vaccinatie bloed afnemen en 4 en 8 weken na vaccinatie. Voor de cellulaire immuniteits bepaling is echter een extra bloedafname 2 weken na vaccinatie nodig. Er wordt dus 4 keer bloed afgenomen, voor vaccinatie, 2, 4 en 8 weken na vaccinatie.

Het totale volume afgenomen bloed in acht weken zal (2x40) + (2x8) = 96ml zijn.

Deze aanpassing zou van toepassing zijn op alle (n=100) proefpersonen die nog geïncludeerd worden.

De vergoeding voor elke deelnemer is (na de laatste bloedafname) 40 euro (10 euro per bloedafname).

**PROEFPERSONENINFORMATIE**

**Gele koorts vaccinatie: vergelijking tussen effectiviteit van subcutane en intracutane injectie zonder viremie bepaling**

Geachte heer/mevrouw,

Graag vragen wij uw medewerking aan het volgende.

Doel en omschrijving van de studie

Het vaccineren tegen de tropische ziekte gele koorts is in veel landen in Midden en Zuid-Amerika en Afrika verplicht voor reizigers. Omdat reeds herhaaldelijke malen een tekort dreigde te ontstaan van de voorraad van dit vaccin, is het belangrijk uit te zoeken of een kleinere dosis van het vaccin een even goede bescherming biedt tegen de ziekte. Om dit te onderzoeken willen we het vaccin in de huid injecteren, in plaats van de huidige methode, onder de huid. Bij andere vaccins heeft deze vaccinatiemethode tot een evengoede immuunrespons geleid.

Om de twee methoden van vaccineren te vergelijken krijgt één groep proefpersonen de vaccinatie in de huid en één groep onder de huid. In welke groep u komt wordt door het toeval bepaald.

Het optreden van bescherming tegen gele koorts na de vaccinatie wordt vastgesteld door bloed af te nemen en daarin de beschermende antistoffen te meten. Er vinden vier bloedafnames plaats, een keer vóór de vaccinatie, twee, vier en acht weken na vaccinatie. Dit zal gebeuren op de vaccinatiepolikliniek van het Leids Universitair Medisch Centrum. Bij de eerste twee bloedafnames wordt 40ml bloed afgenomen (5 buisjes), bij de laatste twee bloedafnames wordt 8ml (1 buisje) bloed afgenomen.

U wordt gevraagd bij te houden of u hinder van de injectiemethode heeft ondervonden. U krijgt hiervoor een dagboekje mee naar huis waarin u kunt aangeven of u klachten heeft en waaruit deze bestaan.

Het is voor de uitkomst van het onderzoek van belang dat u in goede gezondheid verkeert. Dit zal vastgesteld worden aan de hand van een aantal vragen. Het is met name belangrijk dat u geen medicijnen neemt die de afweer onderdrukken.

Te verwachten voordeel van het onderzoek

Het voordeel van dit onderzoek in het algemeen is dat indien intracutane toediening even effectief is als subcutane injectie blijkt, minder vaccin nodig is om reizigers te beschermen tegen gele koorts.

Het persoonlijk voordeel voor u is dat u na dit onderzoek hoogstwaarschijnlijk beschermende afweerstoffen tegen gele koorts virus heeft. Mocht dit niet het geval zijn bieden wij u alsnog het gele koorts vaccin via de huidige vaccinatiemethode aan.

Na derde bloedafname en inlevering van het ingevulde dagboek ontvangt u 40 euro als vergoeding. Indien u het onderzoek voortijdig beëindigd ontvangt u een vergoeding naar rato.

Bijwerking vaccinatie en bloedafname

Bekende bijwerkingen die kunnen optreden bij de huidige methode van vaccineren, onder de huid, zijn lokale reacties: roodheid en zwelling op de injectieplaats. Een paar dagen na de vaccinatie kan spierpijn en lichte koorts optreden. De injectie van het vaccin in de huid kan dezelfde bijwerkingen geven. Bij het afnemen van bloed worden geen ongewenste gevolgen verwacht. Mogelijk krijgt u een blauwe plek op de plaats van injectie.

Verzekering

Er is door het LUMC een verzekering afgesloten waaruit eventuele schade als gevolg van het onderzoek betaald kan worden. Wanneer u vindt dat u schade heeft ondervonden als gevolg van deelname aan het onderzoek kunt u contact opnemen met de arts-onderzoeker. Informatie over de afgeloten verzekering treft u aan in de bijlage.

**Privacy**

Al uw medische gegevens die door de afdeling infectieziekten van het LUMC verzameld worden vallen onder het medisch beroepsgeheim. De uitkomsten van het onderzoek zullen alleen anoniem worden gepubliceerd en zijn nooit te herleiden op naam of geboortedatum. Behalve de onderzoeksmedewerkers heeft niemand inzage in de door u aan ons verstrekte gegevens.

Contact

Als de resultaten uit dit onderzoek leiden tot verdere onderzoeksvragen is het mogelijk dat we graag weer contact met u opnemen, hetzij om u vragen te stellen, hetzij om verder onderzoek te doen. Als u hier bezwaar tegen heeft kunt u dit aangeven op het toestemmingsformulier.

Contactpersonen

De contactpersonen voor dit onderzoek zijn drs. A.H.E. Roukens en dr. L.G. Visser, respectievelijk AIO en internist-infectioloog in het LUMC, werkzaam op de afdeling infectieziekten. Heeft u nog vragen dan kunt u deze aan hen voorleggen. Zij zijn te

bereiken via het secretariaat van de afdeling Infectieziekten van het LUMC op het telefoonnummer 071-5262613.

Indien u behoefte heeft om vragen over dit onderzoek met een onafhankelijke arts te bespreken kunt u contact opnemen met dr. F.P.Kroon, infectioloog in het LUMC, te bereiken op bovenstaand nummer.
