# Supplementary material for: Intradermally Administered Yellow Fever Vaccine at Reduced Dose Induces a Protective Immune Response: A Randomized Controlled Non-Inferiority Trial
Source: PLoS One. 2008 Apr 23;3(4):e1993. doi: 10.1371/journal.pone.0001993 (PMC2297511; doi:10.1371/journal.pone.0001993)
Supplement: Protocol S3 — Trial Protocol (0.03 MB DOC) [file pone.0001993.s004.doc]

**Amendement 2**

**Protocol P05.059**

5 september 2006

Titel: Gele koorts vaccinatie: vergelijking tussen effectiviteit van subcutane en intracutane injectie

Dit amendement betreft een verzoek om het onderzoeksprotocol uit te breiden met een bloedafname 1 jaar na de gele koorts vaccinatie.

Voorlopige resultaten van dit onderzoek suggereren dat de intracutane een evengoede bescherming biedt als de subcutane vaccinatie. Om te onderzoeken of deze immuunresponsen tengevolge van de verschillende toedieningswegen evenlang stand houden willen wij bij de proefpersonen die reeds geïncludeerd en gevaccineerd zijn 1 jaar na vaccinatie bloed afnemen. Hierbij wordt 1 buisje bloed (8ml) afgenomen.

Gezien per bloedafname een vergoeding van 10 euro geldt, wordt dit bedrag aan deze deelnemers verstrekt.

**PROEFPERSONENINFORMATIE**

**Gele koorts vaccinatie: vergelijking tussen effectiviteit van subcutane en intracutane injectie, 1 jaar na vaccinatie**

Geachte heer/mevrouw,

Graag vragen wij uw medewerking aan het volgende.

Ongeveer 1 jaar geleden heeft u deelgenomen aan het onderzoek waarbij verschillende vaccinatie methoden voor gele koorts vaccinatie werden vergeleken. Omdat wij de immunologische respons 1 jaar na de vaccinatie willen meten, is het nodig 1 buisje bloed (8ml) af te nemen. Bij deelname aan het gele koorts onderzoek van 1 jaar geleden heeft u ons toestemming gegeven contact met u op te nemen.

**Te verwachten voordeel**

Uw bijdrage aan dit onderzoek kan een belangrijke wetenschappelijke bijdrage leveren. Daarnaast ontvangt u voor deze bloedafname 10 euro.

Bijwerking bloedafname

Bij het afnemen van bloed worden geen ongewenste gevolgen verwacht. Mogelijk krijgt u een blauwe plek op de plaats van injectie.

**Privacy**

Al uw medische gegevens die door de afdeling infectieziekten van het LUMC verzameld worden vallen onder het medisch beroepsgeheim. De uitkomsten van het onderzoek zullen alleen anoniem worden gepubliceerd en zijn nooit te herleiden op naam of geboortedatum. Behalve de onderzoeksmedewerkers heeft niemand inzage in de door u aan ons verstrekte gegevens.

Contact

Als de resultaten uit dit onderzoek leiden tot verdere onderzoeksvragen is het mogelijk dat we graag weer contact met u opnemen, hetzij om u vragen te stellen, hetzij om verder onderzoek te doen. Als u hier bezwaar tegen heeft kunt u dit aangeven op het toestemmingsformulier.

Contactpersonen

De contactpersonen voor dit onderzoek zijn drs. A.H.E. Roukens en dr. L.G. Visser, respectievelijk AIO en internist-infectioloog in het LUMC, werkzaam op de afdeling infectieziekten. Heeft u nog vragen dan kunt u deze aan hen voorleggen. Zij zijn te

bereiken via het secretariaat van de afdeling Infectieziekten van het LUMC op het telefoonnummer 071-5262613.

Indien u behoefte heeft om vragen over dit onderzoek met een onafhankelijke arts te bespreken kunt u contact opnemen met dr. F.P.Kroon, infectioloog in het LUMC, te bereiken op bovenstaand nummer.
