# Supplementary material for: Intradermally Administered Yellow Fever Vaccine at Reduced Dose Induces a Protective Immune Response: A Randomized Controlled Non-Inferiority Trial
Source: PLoS One. 2008 Apr 23;3(4):e1993. doi: 10.1371/journal.pone.0001993 (PMC2297511; doi:10.1371/journal.pone.0001993)
Supplement: Protocol S4 — Trial Protocol (0.03 MB DOC) [file pone.0001993.s005.doc]

**Addendum**

**Protocol P05.059**

25 oktober 2007

Auteurs: Drs. A Roukens/Dr. LG Visser

*betreffende*

Gele koorts vaccinatie: vergelijking tussen effectiviteit van subcutane en intracutane injectie

Dit addendum betreft een verzoek tot goedkeuring voor een inclusiestop gebaseerd op een gecorrigeerde berekening na interim-analyse van tweemaal 50 individuen. Conform het protocol vond na inclusie van tweemaal 50 vrijwilligers een tussenanalyse plaats. Hierbij bleek dat zowel na subcutane als intracutane injectie bij alle individuen in beide onderzoeksarmen seroprotectie (d.i., tweemaal 50/50) optrad (samenwerking met dr. R. Brand, Medische Statistiek LUMC). Dit was aanleiding tot herevaluatie van de noodzaak om nog tweemaal 150 individuen te gaan includeren, zoals gesuggereerd in het oorspronkelijke protocol.

De berekening van de populatiegrootte om (eenzijdige) non-inferioriteit aan te tonen was bij aanvang, zo bleek na interim-analyse na inclusie van 50 deelnemers per interventiegroep, onjuist.

De hernieuwde berekening van de te includeren populatie bleek bij een β van 0.8 en α van 0.05, verwacht succes in beide groepen van 0.99 en een verschil van 0.04, 77 per groep (Armitage et al., 4th edition, 2002, formule 18.5).

Deze berekening wordt ondersteund door R. Wolterbeek (Medische Statistiek LUMC) in bijl. 1. Hij berekent 78 deelnemers per groep door de β van 0.81.

Inmiddels zijn in de controle groep 78 en in de interventie groep 77 deelnemers geïncludeerd, en is op basis van de hernieuwde berekening gestopt met de inclusie van deelnemers.

Voor viremiebepaling zijn, zoals beschreven in het protocol, 40 deelnemers geincludeerd. Twintig hiervan in de groepen die hierboven zijn beschreven (10 in de experimentele en 10 in de controlegroep) en 20 deelnemers die eerder gevaccineerd waren tegen gele koorts.
